# Cavity Lasing Characteristics of Thioflavin T and Thioflavin X in Different Solvents and Their Interaction with DNA for the Controlled Reduction of a Light Amplification Threshold in Solid-State Biofilms

**DOI:** 10.1021/acsaom.3c00264

**Published:** 2023-10-05

**Authors:** K. Rusakov, S. Demianiuk, E. Jalonicka, P. Hanczyc

**Affiliations:** † Institute of Experimental Physics, Faculty of Physics, University of Warsaw, Pasteura 5, 02-093 Warsaw, Poland; ‡ Faculty of Construction and Environmental Engineering, Warsaw University of Life Sciences, 02-776 Warsaw, Poland

**Keywords:** Thioflavin T, Thioflavin X, lasing, Fabry−Perot cavities, DNA, aggregation, sonication

## Abstract

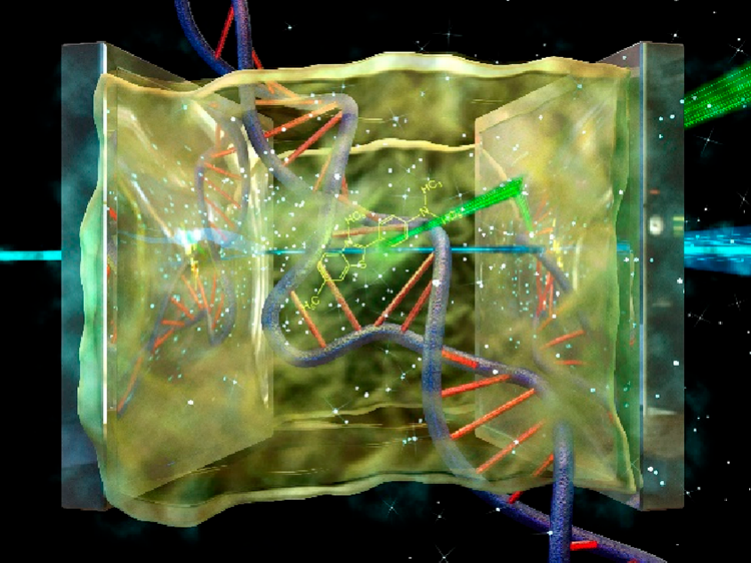

The lasing characteristics of Thioflavin T (ThT) and Thioflavin X (ThX) dyes were investigated in solvents with increasing viscosity: water, ethanol, butanol, ethylene glycol, and glycerol and three forms of DNA (double-helix natural, fragmented, and aggregated). The results identified that lasing thresholds and photostability depend on three critical factors: the solvation shell surrounding dye molecules, the organization of their dipole moments, which is driven by the DNA structure, and the molecules diffusion coefficient in the excitation focal spot. The research highlights that dye doped to DNA accumulated in binding sites fosters long-range dye orientation, facilitating a marked reduction of lasing thresholds in the liquid phase as well as amplified spontaneous emission (ASE) thresholds in the solid state. Leveraging insights from lasing characteristics obtained in liquid, ASE in the solid state was optimized in a controlled way by changing the parameters influencing the DNA structure, i.e., magnesium salt addition, heating, and sonication. The modifications led to a large decrease in the ASE thresholds in the dye-doped DNA films. It was shown that the examination of lasing in cavities can be useful for preparing optical materials with improved architectures and functionalities for solid-state lasers.

Thioflavin dyes are small organic fluorophores that belong to the group of molecular rotors.^1,2^ A well-known representative from this group is Thioflavin T (ThT), which is extensively used in the field of protein aggregation associated with neurodegenerative diseases.^3,4^ Another notable member of this group is Thioflavin X (ThX), which, like ThT, exhibits unique photophysical properties influenced by its environment.^5^ A defining characteristic of ThT, ThX, and other molecular rotors is their low fluorescence quantum yield in low-viscosity environments.^6^ Changes in the environment’s viscosity or binding to biomolecules, such as amyloid protein fibrils^7−9^ or DNA,^10−12^ results in significant emission enhancement. The underlying photophysical mechanism for this fluorescence boost is related to ultrafast torsional motion leading to nonradiative deactivation via twisted intramolecular charge transfer (TICT).^13^ When the internal rotation of its molecular segments is hindered, either in high-viscosity solvents like glycerol or due to binding to biomolecules such as DNA, there is a dramatic increase in the fluorescence signal.^14^

Recently, it was found that complete inhibition of ThT molecules in solid-state films creates favorable conditions for population inversion and light amplification that is named amplified spontaneous emission (ASE).^15^ The physical manifestation of ASE in solid films can be observed when the excitation energy is gradually increased (Figure 1a).^16^ At a given energy, the emission spectrum becomes significantly narrower and a significant rise of the light intensity could be observed (ASE marked in red in Figure 1a).^17^ The pump energy at which that happens is named the light amplification threshold (or ASE threshold). The same principle works in the cavity lasing.^18^ However, unlike ASE measurements in solid films, where micro- or nanoresonators are formed in a spontaneous process upon drying, the Fabry–Perot cavity lasing has two mirrors that act as photonic resonators. A practical difference between the two experiments is the configuration for collecting the signal from the sample. In films, ASE is collected perpendicular to the excitation beam, and in cavity lasing, it is detected in parallel to the excitation beam (Figure 1b,c). In the latter experiment, the excitation beam is cut off with the appropriate filter, and only the lasing signal from the sample is collected (Scheme S1). In films, ASE and fluorescence signals are simultaneously collected (images of rising fluorescence and the appearance of an intense ASE spot are shown in Figure 1, left inset). In the cavity when the threshold for population inversion is reached, a narrow lasing peak is detected without background fluorescence. The full width at half-maximum of the lasing spectrum in cavities is only a few nanometers wide, whereas the ASE in films is between 10 and 20 nm wide.

**Figure 1:**
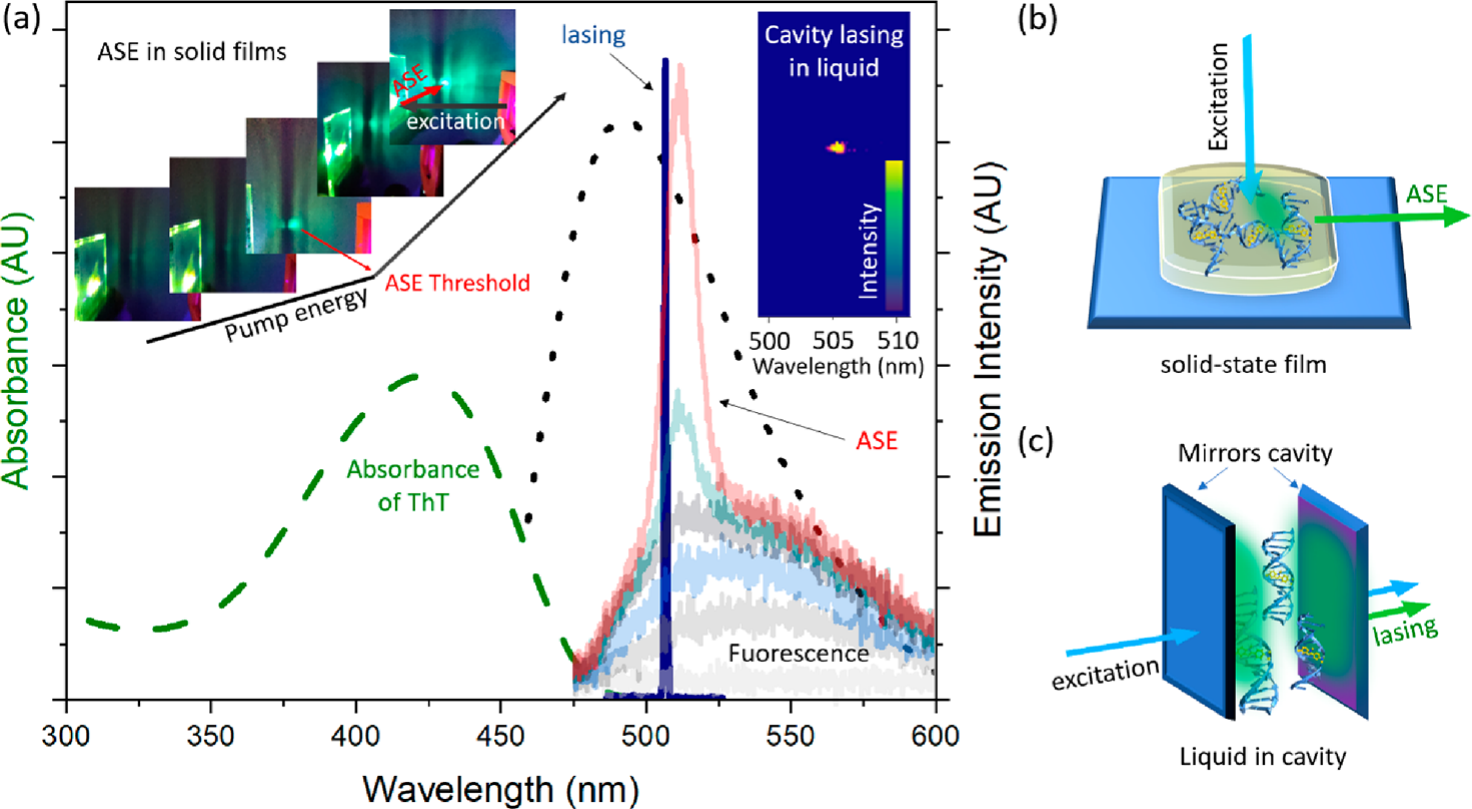
(a) Absorption (green dashed), emission (black dotted), ASE in solid films (a signal rise shown from light gray to red), and lasing (dark-blue solid) of ThT in glycerol. The insets represent examples of the measured solid-state films (left inset) and the lasing signal in the mirror cavity, with the bright spot being the lasing effect arising in ThT when crossing the lasing threshold (right inset). (b) Cartoon representing the configuration for ASE measurements in films. (c) Drawing of the mirror cavity lasing configuration, with sandwiched liquid containing DNA and ThT that acts as a gain medium. Solid-state samples were excited at 400 nm, and liquid samples in cavities were excited at 430 nm.

The mirror cavity provides strong optical feedback that can be used to detect subtle molecular changes in the gain medium.^19,20^ The advantage of the cavity lasing is also the possibility of studying biomolecules in liquid, which is more convenient than a solid phase for elucidating the influence of external factors on the biomolecule structure. In the case of dye-stained biomolecules, fluorophores act as gain materials in the cavity and are providing information on the biomolecule structure. Thus, the combination of cavity lasing in liquid and ASE in solid films is a first example of the structural tuning of a DNA–dye complex in solution for processing optimized biofilms with improved optical properties.

Improving the optical properties of organic materials in the solid state is a major challenge in the field of organic lasers.^21^ Usually, a high current is necessary to achieve population inversion in dye lasers. This not only poses a technological hurdle but also raises concerns about the device efficiency and longevity. In this context, DNA hosting dyes offer a promising avenue. The DNA–dye approach is not limited to any specific type of dye and can easily be generalized to a wide range of organic materials. This opens up possibilities for the development of new types of organic lasers with customized properties driven by the DNA structure.

DNA staining with ThT was refreshed after the discovery of strong fluorescence enhancement in genetically important structures: G-quadruplexes and i-motifs^22^ that are linked with genetic and cancer diseases. It is noteworthy that only moderate emission enhancement in double-stranded DNA was reported, but it was not studied in detail.^23^ Choosing ThT and ThX allows for a deeper understanding of the lasing characteristics of the important class of molecular rotors and exploration on the general concept of DNA–dye complexes for designing materials with enhanced optical properties. Recently, it was found that ThT can bind to DNA by intercalation.^24^ This particular binding mode led to the alignment of dye molecules with respect to each other.^25^ In terms of lasing and ASE generation, this means the alignment of electronic transition-dipole moments, which can significantly reduce the lasing threshold compared to the system of randomly oriented molecules. DNA’s ability to form various structural motifs (duplexes, triplexes, quadruplexes, etc.) offers a wide range of options for tuning the optical properties when combined with dyes.

In this paper, we examine the lasing of ThT and ThX dyes in five solvents with increasing viscosity (water, ethanol, butanol, ethylene glycol, and glycerol) in order to characterize the light amplification mechanism in two representative dyes of the molecular rotor family (Figure 2). Next, the two dyes were investigated in the presence of a DNA duplex in three forms: a natural native long DNA duplex from calf-thymus, a fragmented DNA by ultrasonication, and an aggregated form that was prepared using magnesium salt and quaking (for details of the sample preparation and a schematic illustration of the lasing setups, see Schemes S1 and S2).

**Figure 2:**
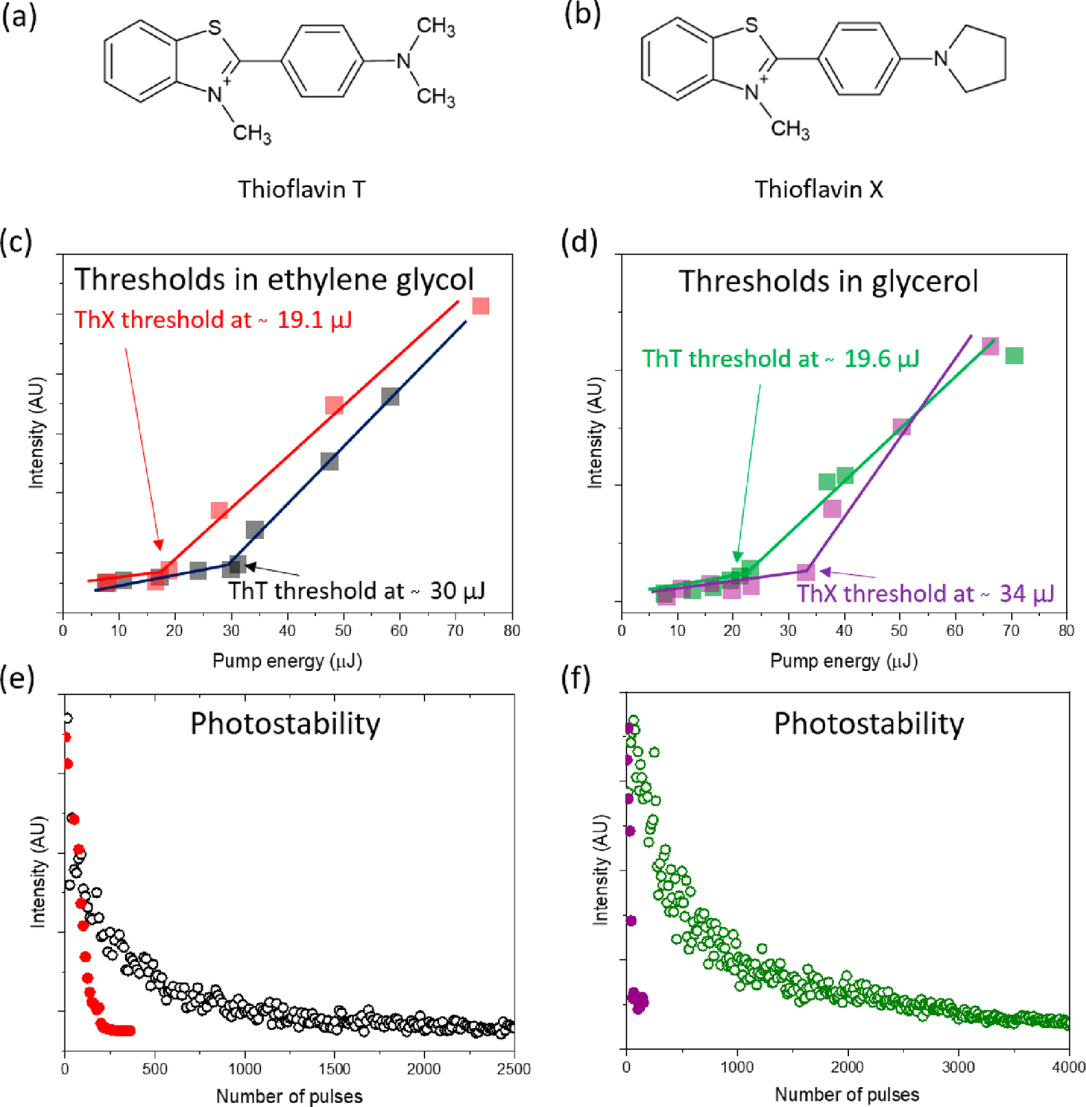
(a) Structure of ThT. (b) Structure of ThX. (c) Lasing thresholds of ThT (black) and ThX (red) in ethylene glycol. (d) Lasing thresholds of ThT (green) and ThX (violet) in glycerol. Dye photobleaching decays: (e) In ethylene glycol, ThT photobleaching is presented as black open circles and ThX is presented as red dots. (f) In glycerol, the photobleaching of ThT is shown as green open circles and ThX as violet dots. Samples were excited at 430 nm.

The purpose of cavity lasing experiments was to characterize the ThT and ThX light amplification properties and examine different forms of DNA in the liquid phase. It was shown previously that lasing signals are sensitive to changes of the DNA structure.^26^ In this paper, an optimized DNA–dye system in the liquid phase was used to form a solid-state biofilm, whereby ASE thresholds were reduced in a controlled way by tuning DNA–dye interactions with thermal treatment, sonication, and counterions. Such an approach allows one to make more efficient optical materials with significantly improved ASE characteristics.

Because it is well-known that one of the parameters that strongly affects the fluorescence quantum yields of ThT and ThX is viscosity, the dyes were dissolved in solvents with rising viscosity (Table 1) in order to verify whether the lasing effect can be detected and in which solvents. It was found that there is no lasing in water. ThT lasing was detected in four other solvents (ethanol, butanol, ethylene glycol, and glycerol), whereas ThX lasing was detected only in ethylene glycol and glycerol. For each particular solvent, the dye spectrum and photostability were measured and thresholds were determined based on three consecutive measurements (Figure 2). The studies revealed that dye–solvent interactions are critical for the lasing effect and its photostability. As expected, ThT lasing thresholds were lower when viscosity was higher in the following order: ethanol (highest threshold) → butanol → ethylene glycol → glycerol (lowest threshold). Surprisingly, the opposite effect was observed for ThX, whereby lasing was detected only in ethylene glycol and glycerol, with a lower lasing threshold in a lower-viscosity solvent. The possible explanation could be related to the polarity.

**Table 1:** Absorbance and Emission Maxima of ThT and ThX in Five Solvents (Water, Ethanol, Butanol, Ethylene Glycol, and Glycerol) and Summary of the Results on Lasing Thresholds and Photostability in Various Solvents^a^

		λ_abs_ (nm)		λ_em_ (nm)			lasing thresholds (μJ)		photostability (number of pulses)	
	viscosity^b^ (×10^–3^ Pa s)	ThT	ThX	ThT	ThX	dye concentration for lasing (mM)	ThT	ThX	ThT	ThX
water	0.89	412	419	475	490	78.4				
ethanol	1.08	418	424	486	494	25	73.6		>5000	
butanol	2.59	419	424	487	495	25	32.7		>5000	
ethylene glycol	16.1	420	426	490	499	18.8	30	19.1	∼1000	∼400
glycerol	934	424	430	493	502	3.14	19.6	34	∼3000	∼120

a Lasing experiments were carried at 430 nm.

b Solvent viscosity values: http://murov.info/orgsolvents.htm.

The influence of the polarity on the lasing thresholds can be analyzed when looking at two solvent pairs, ethanol/butanol and ethylene glycol/glycerol, for ThT and a single pair, ethylene glycol/glycerol, for ThX. The polarity can be interpreted by the dielectric constant (ε) of the solvents. Overall, the higher the ε, the more polar the solvent (ethanol, ε ∼ 25; butanol, ε ∼ 17.3; ethylene glycol, ε ∼ 37.7; glycerol, ε ∼ 42.5).^27^ Taking into account that ethanol is more polar than butanol and the ThT lasing threshold is significantly lower in less polar butanol, this means that the polarity is of lesser importance for ThT lasing than the viscosity. A further significant decrease in ethylene glycol and glycerol, which have similar ε values, confirms that the viscosity is the major factor for lasing in ThT. The opposite is true for ThX, whereby the threshold is lower in less polar ethylene glycol than in glycerol. Ethylene glycol is also a significantly less viscous solvent than glycerol. Thus, in the case of ThX, both the viscosity and polarity are important factors in generating lasing.

Next, the two dyes were examined in the context of photodegradation at a fixed pump energy of 110 μJ. The lasing signal in ThT was extremely stable in butanol, and degradation was gradually decreasing in a linear trend in ethanol (Figure S1). Photodegradation in the two remaining solvents (ethylene glycol and glycerol) is shown in Figure 2e,f, and lasing was detected for up to 1000 and 3000 excitation pulses, respectively. The trend is biexponential with fast degradation at the beginning of the process and a slower rate in the next stage. The results indicate that the photostability is related to dye–solvent interactions and the rate of excited-state energy release to the solvent before complete fluorophore decomposition. The second reason is the molecule diffusion coefficient in liquid, whereby in low-viscosity solvents, the motion of molecules is faster and photodegraded fluorophores can be quickly replaced in the excitation focal point. In contrast, a high viscosity slows dye motion, and molecules in the excitation focal point were degraded and not replaced in the diffusion process (Table 1).

The second dye–ThX that showed lasing only in ethylene glycol and glycerol was photobleached after 100 and 400 pulses, respectively. In comparison to ThT in the same solvents, ThX is degraded relatively fast. The reason could be the chemical replacement of the methyl group by pyrrolidine, which restricts rotation around the C(sp^3^)–N σ bonds and increases the electron density on the benzothiazole ring. That change of the molecular structure of the fluorophore causes an accumulation of high-energy pulses, which leads to rapid chemical decomposition. It is noteworthy that extending the conjugation system with pyrrolidine may also impact diffusion, which can be a reason for faster ThX photobleaching than in the case of a smaller ThT molecule.

In the next step, lasing thresholds and photobleaching of dyes were examined in the presence of DNA, taking into account DNA and dye concentrations as well as applying certain temperatures to the cavity containing the gain material.

Figure 3a shows lasing thresholds with changing dye concentrations, whereby the DNA content was fixed at *C*_DNA_ = 20 mM. It was found that, in the range *C*_ThT_ = 10–15 mM, the thresholds were visibly lower than those in the range *C*_ThT_ = 17–26 mM. This indicates that, above a certain dye concentration, the binding sites are saturated and there are a large number of unbound fluorophores. Studies on free molecules showed that millimolar concentrations cause energy transfers by the fluorescence resonance energy transfer (FRET) mechanism, that is, quenching fluorescence. The FRET efficiency is concentration-dependent, and the emission is decreasing linearly with increasing dye content.^28^ To confirm that also the FRET mechanism arising from free dye is quenching lasing induced in DNA bound molecules, we measured lasing at the highest studied dye concentration *C*_ThT_ = 26 mM (Figure 3b). Starting from *C*_DNA_ = 18 mM, we observed a gradual reduction of lasing thresholds with increasing DNA concentration. The trend was linear and similar to that of concentration-dependent quenching studies performed in free fluorophores. The linear trend indicates that, at such high dye contents, all possible DNA binding sites (intercalation, groove binding, and external binding) are immediately filled and lasing is solely dependent on quenching arising from the ratio between bound and unbound dye molecules. First, less DNA means that a smaller number of bound dyes contribute to lasing. Second, free fluorophores that are not lasing additionally quench lasing of DNA bound molecules by the FRET mechanism. As a consequence, a higher pump energy is required to reach the lasing threshold.

**Figure 3:**
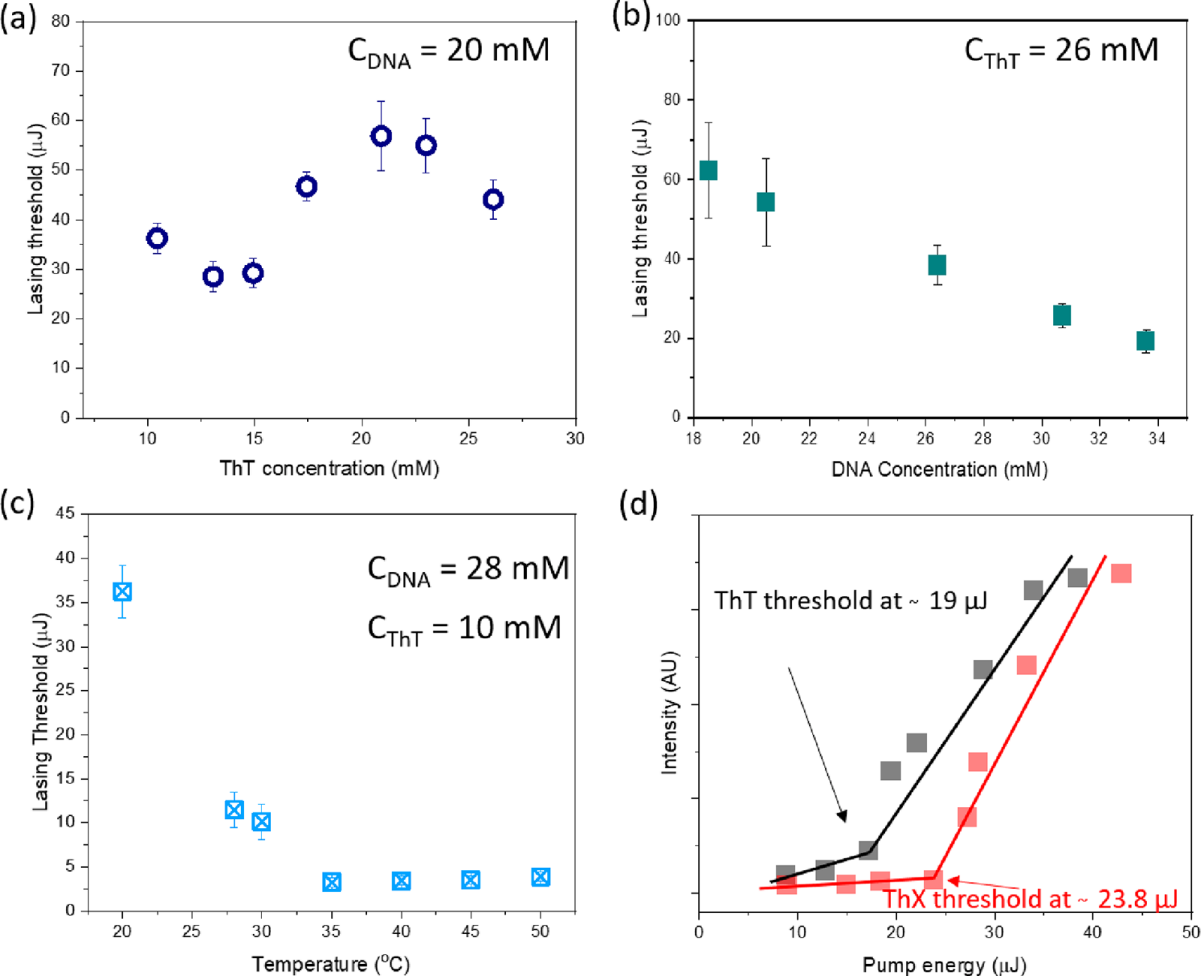
(a) Lasing thresholds in function of ThT concentration. The minimum dye concentration required to obtain lasing was *C*_ThT_ = 10 mM with the DNA concentration fixed to *C*_DNA_ = 20 mM. (b) Lasing thresholds in changing DNA concentrations. The minimum DNA concentration required to obtain lasing was *C*_DNA_ = 18 mM with dye fixed at *C*_ThT_ = 26 mM. (c) Changes of the lasing thresholds during DNA heating from room temperature to 50 °C that cause partial unwinding of the DNA helix. (d) Example of thresholds determined for ThT (black) at *C*_ThT_ = 26 mM and *C*_DNA_ = 34 mM and ThX (red) at *C*_ThX_ = 26 mM and *C*_DNA_ = 34 mM in the presence of DNA. Samples were excited at 430 nm.

In the next step, DNA with 10 mM dye content was stepwise heated from 25 to 50 °C. Partial unwinding of DNA in elevated temperature provides better access for dye molecules to slide into the intercalation pockets. Figure 3c shows that already in the range 28–30 °C there is a dramatic decrease of lasing thresholds due to additional dye binding. Another substantial reduction of the lasing threshold to 3.5 μJ occurs around 35 °C. Making a full cycle to 50 °C and then lowering the temperature back to 25 °C reduces the pumping fluence required for population inversion to 2.5 μJ. This result means that still a lot of free fluorophores are present in the system even at *C*_ThT_ = 10 mM before heating. Partial unwinding of the DNA helix allows to accommodate more dye molecules between the DNA strands, which has a significant impact on the lasing parameters and especially on the lasing thresholds. The main reason is the alignment of electronic transition-dipole moments of dye molecules in the intercalation pockets and the higher probability of lasing at lower pumping energy than in a system of randomly oriented molecules. The probability of inducing lasing in one molecule by a photon spontaneously emitted by another molecule is the highest for molecules with mutually parallel transition moments. Thus, the lowest lasing threshold measured for ThT with DNA may reflect the local order of intercalated dye molecules.

The lasing characteristics of ThX in the presence of DNA were similar to that of ThT with slightly higher lasing thresholds (Figure 3d). Slightly higher thresholds could be an indication that ThX, which has pyrrolidine group in its chemical structure, has a lower affinity to the intercalation sites due to steric hindrance. However, unwinding the DNA helix in the temperature experiments reduces the lasing threshold of ThX to levels similar to those detected in ThT, meaning that the ThX analogue also intercalates the DNA duplex.

Next, the lasing experiments were carried out with DNA in two forms: fragmented DNA obtained by ultrasonication^29^ and aggregated DNA obtained in the process of melting and quick annealing with quaking in the presence of divalent magnesium chloride.^30^Figure 4 shows the results on the lasing thresholds and the photobleaching of both dyes in the presence of different forms of DNA.

**Figure 4:**
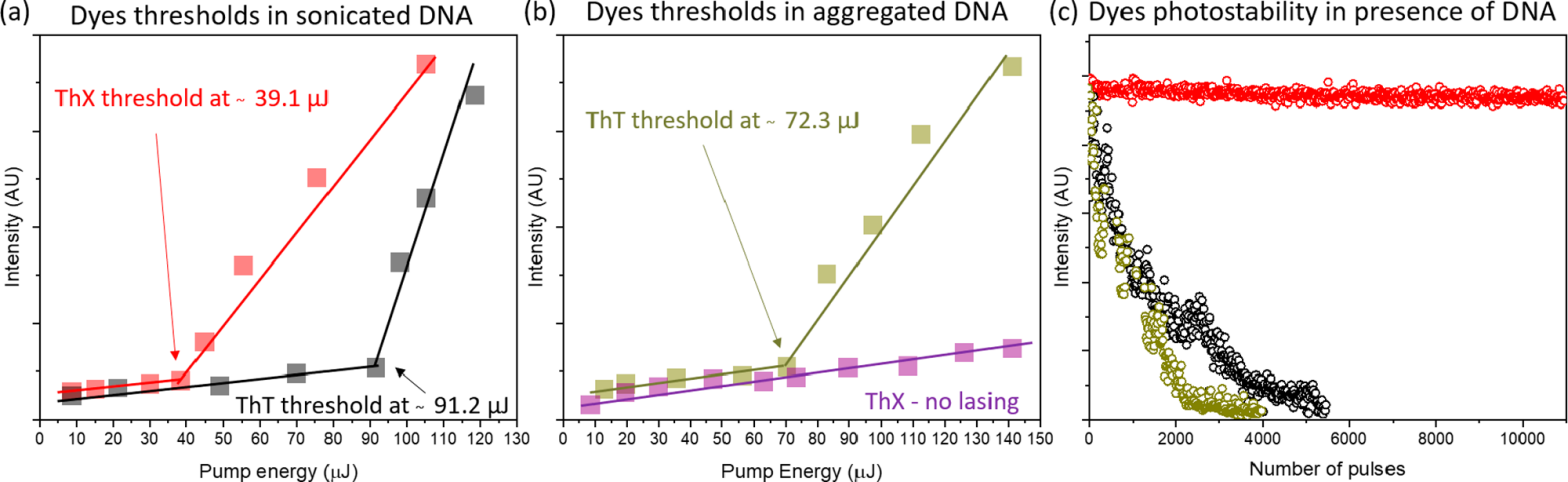
(a) Lasing thresholds of ThT (black) and ThX (red) in the presence of sonicated DNA. (b) Lasing threshold of ThT (dark yellow) in the presence of aggregated DNA. No lasing was detected for ThX dye (violet). (c) Photostability decays of ThT in the presence of sonicated DNA (black dots) and in the presence of aggregated DNA (dark-yellow dots) and photostability of ThX in the presence of sonicated DNA (red dots). *C*_ThT_ = 26 mM, *C*_ThX_ = 26 mM, and *C*_DNA_ = 34 mM. Samples were excited at 430 nm.

In sonicated and aggregated DNA, the lasing characteristics of the dyes differ significantly from those observed with untreated DNA. Specifically, the lasing thresholds are noticeably higher in dye-doped sonicated and aggregated DNA compared to untreated DNA. For ThT, the lasing thresholds increase from 19 μJ in untreated DNA to 91.2 μJ in sonicated DNA and 72.3 μJ in aggregated DNA, while ThX requires nearly twice the pumping fluence (39.1 μJ) in sonicated DNA compared to that in untreated DNA (23.8 μJ). Notably, in the case of aggregated DNA, ThX lasing was not observed, even at the highest pump energy levels.

In the case of the rise of thresholds in sonicated DNA, the advantageous long-range orientation of dye molecules is disrupted when DNA undergoes sonication, resulting in a higher pumping fluence needed to reach the lasing threshold. That was confirmed in the control experiment whereby lasing thresholds were determined after 5 s periods of sonication until the lasing signal was no longer detected after 2 min (Figure S2).

In contrast to ThT, ThX displayed lower lasing thresholds compared to ThT in sonicated DNA, and no lasing was observed in aggregated DNA (Figure 4a,b). The lower lasing threshold of ThX compared to ThT in sonicated DNA can be explained only by using the photobleaching results. To our surprise, ThX showed excellent photostability in the presence of short fragments of DNA (red line in Figure 4c). That is outstanding when recalling the photostability of pristine ThX in ethylene glycol or glycerol, whereby ThX was degraded after a few hundred pulses (Figure 2e,f). In the presence of untreated DNA, degradation was even more pronounced and ThX photobleached after a few excitation pulses (the results are not shown). However, sonication of DNA to short fragments causes a dramatic stabilization of the lasing signal of ThX (ThX stability > 10000 pulses). A similar effect was only observed in pristine ThT dissolved in butanol (Figure S1). Those results show that, for lasing in liquids, diffusion is also an important factor. The importance of diffusion was confirmed in ThT mixed with sonicated small DNA fragments and large DNA aggregates. In sonicated DNA, ThT photobleached completely after approximately 5000 pulses, whereas in aggregated DNA, dye was degraded after 3500 pulses. Fragmented DNA was being replaced faster in the excitation focal point than large DNA aggregates, which affected the overall photostability of the dyes.

Characterization of ThT–DNA lasing in cavities helped to prepare optimized materials with low ASE thresholds in the solid state. Solutions prepared for cavity lasing were drop-casted on glass slides and left for drying, and solid films were tested in the experimental configuration for ASE generation in the solid state (Figure 1b). This synergistic approach of combining cavity lasing in liquid with ASE in solid films represents the advancement in optimizing DNA-based materials for lasing application. The results presented in the cavities show that dual methodology allows for unprecedented structural tuning of the DNA–dye complexes in solution, creating a well-defined protocol for preparing biofilms with better ASE characteristics in the solid state.

The key finding was that films made of sonicated and heated DNA with magnesium salt allowed us to lower the pump fluence for ASE generation in solid-state samples by more than 50%. Figure 5 represents the ASE thresholds measured in ThT- and ThX-stained DNA films. Table 2 summarizes the ASE threshold results for the tested drop-casted solid-film samples.

**Figure 5:**
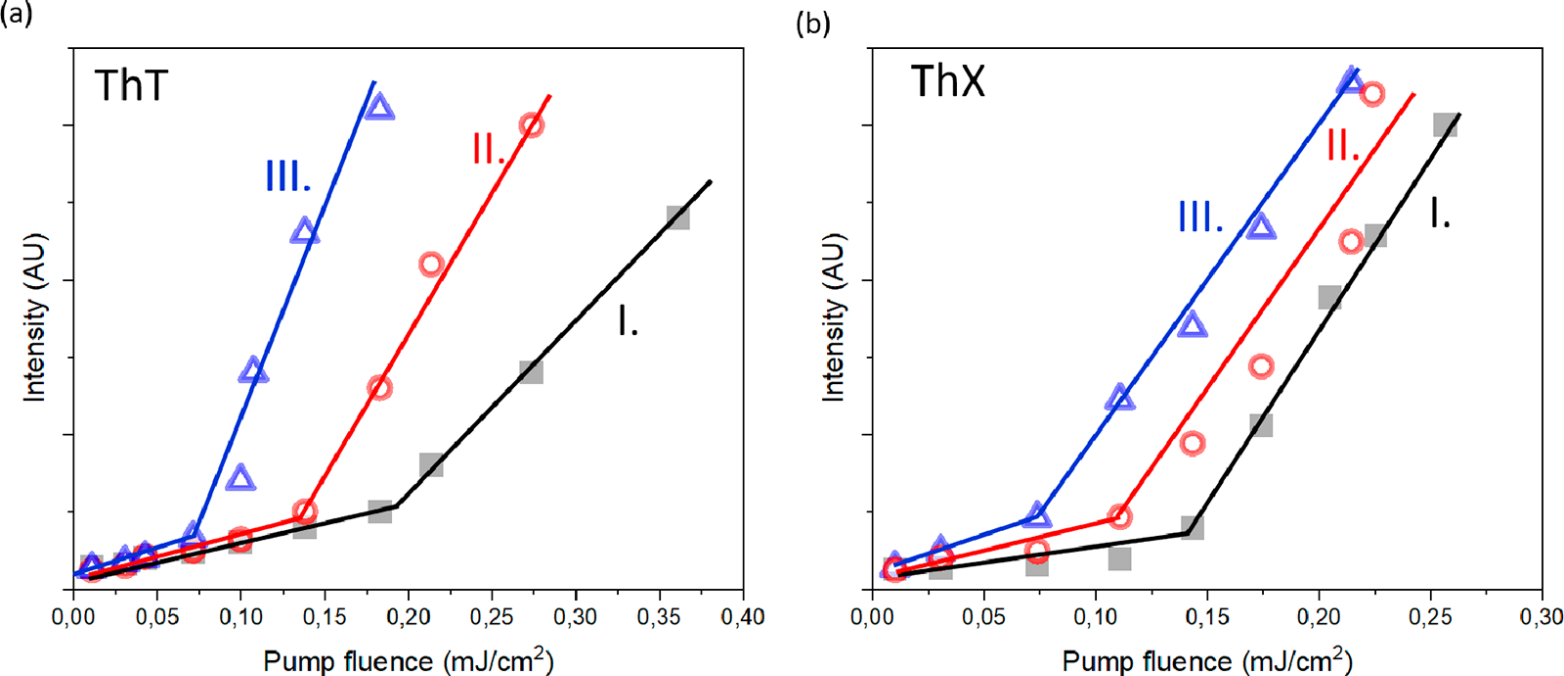
ASE thresholds in DNA stained with (a) ThT and (b) ThX. Markings correspond to (I) untreated calf-thymus DNA (black color), (II) untreated calf-thymus DNA in the presence of magnesium salt (red color), and (III) DNA sonicated and heated in 50 °C and then slowly cooled to 25 °C in the presence of magnesium salt (blue color). Solid films were prepared of *C*_DNA_ = 40 mM and *C*_dye_ = 10 mM.

**Table 2:** ASE Thresholds of Drop-Casted Films of ThT- and ThX-Stained DNA^a^

	ASE threshold (mJ/cm^2^)	
	ThT	ThX
untreated DNA	0.195 ± 0.01	0.140 ± 0.01
untreated DNA + MgCl_2_	0.140 ± 0.01	0.120 ± 0.01
heated DNA + MgCl_2_	0.090 ± 0.01	0.110 ± 0.01
heated DNA + MgCl_2_ + sonication	0.070 ± 0.005	0.075 ± 0.01

a For each solution sample before drop casting, the DNA concentration was equal to 40 mM and the ThT concentration was equal to 10 mM.

When the ASE thresholds in films made of pure DNA stained with dyes were compared to those with biofilm containing 10 mM magnesium salt, there was a significant decrease of the ASE threshold in the presence of magnesium ions (Figure 5). The ASE thresholds were reduced from 0.195 to 0.140 mJ/cm^2^ in the case of ThT-stained DNA films and from 0.140 to 0.120 mJ/cm^2^ for ThX–DNA films. Lower thresholds in ThX than in ThT can be explained by a higher extinction coefficient, meaning that the energetic barrier for achieving the population inversion is lower.

Another sample was prepared by heating a dye–DNA solution with magnesium salt in 50 °C. As the cavity lasing experiments show, heating unwinds the DNA helix and allows more ThT molecules to enter between DNA strands. Ordering molecules in the intercalation pockets causes the dye transition-dipole moments to be oriented parallel with respect to each other and the ASE threshold in that samples were equal to 0.09 mJ/cm^2^ for ThT–DNA films and 0.11 mJ/cm^2^ for ThX–DNA films.

Sonication for 5 s chopped the DNA into shorter fragments. It is well-known that shorter DNA is more structurally rigid. As the lasing in liquids shows, a short sonication time has a minimal influence on the lasing thresholds. However, shorter DNA–dye fragments can organize easier upon drying the drop-casted film. The lowest ASE thresholds for both dyes, ThT and ThX, were obtained for DNA films that were dissolved in the presence of magnesium salt, sonicated for 5 s, heated in 50 °C for 10 min, and then slowly annealed.

The results presented show that DNA as a host for dyes offers a compelling solution because it provides a stable yet flexible framework that can host a variety of dye molecules, facilitating efficient conditions for light amplification. DNA structural adaptability paves the way for the development of materials for solid-state lasers, where the optical properties can be customized based on the structural attributes of DNA.

The results show that DNA structural adaptivity significantly lowers the energy inputs required for ASE generation, thereby potentially addressing the issues associated with the efficient reduction of thresholds for solid-state lasers pumped by an electrical current. The traditional challenges in solid-state organic lasers, such as the need for high currents to achieve population inversion, are well-known bottlenecks. The combination of the cavity lasing in liquid and ASE in the solid state can be helpful in optimizing the functionality of dyes, for example, in DFB lasers.

In summary, the lasing parameters of ThT and ThX were characterized in solvents ethanol, butanol, ethylene glycol, and glycerol in terms of the thresholds for obtaining population inversion in cavities and the photodegradation of dyes. Next, lasing of fluorophores was examined in the presence of DNA in its native untreated form, after DNA fragmentation and after DNA aggregation. Lasing measurements with DNA revealed three factors to be critical for ThT and ThX as gain media. First, the type of interaction with DNA is particularly important to reduce the lasing thresholds. Second, the molecule diffusion coefficient in liquid determines the rate of fluorophore replacement in the excitation focal spot. Third, the chemical structures of the dyes and the dye–solvent interaction are also important. Characterization of the lasing in liquids helped to optimize the DNA–dye system for light amplification in the solid state. Magnesium salt, heating and sonication of DNA cause a strong reduction of the ASE threshold in dye-doped DNA films.

## Supporting Information Available

Materials, preparation protocols, and method descriptions (PDF)
