# Supplementary material for: Cavity Lasing Characteristics of Thioflavin T and Thioflavin X in Different Solvents and Their Interaction with DNA for the Controlled Reduction of a Light Amplification Threshold in Solid-State Biofilms
Source: ACS Appl Opt Mater. 2023 Oct 5;1(12):1922–9. doi: 10.1021/acsaom.3c00264 (PMC10749465; doi:10.1021/acsaom.3c00264)
Supplement: Supplementary file 1 — ot3c00264_si_001.pdf [file ot3c00264_si_001.pdf]

SUPPORTING INFORMATION

# Cavity Lasing Characteristics of Thioflavin T and Thioflavin X in Different Solvents and Their Interaction with DNA for Controlled Reduction of Light Amplification Threshold in Solid-State Biofilms

K. Rusakov[1,2], S. Demianiuk[1], E. Jalonicka[1], P. Hanczyc[1*]

[1] Institute of Experimental Physics, Faculty of Physics, University of Warsaw, Pasteura 5, 02-093 Warsaw, Poland

[2] Faculty of Construction and Environmental Engineering, Warsaw University of Life Sciences, 02-776, Warsaw, Poland

Correspondence to: *piotr.hanczyc@fuw.edu.pl

## Materials and methods

**Thioflavin T** Ultrapure grade Thioflavin T (ThT) was purchased from AATBioquest. A stock solution for lasing experiments in cavities was prepared in distilled water at a concentration of 78.4 mM.

**Thioflavin X** was synthesized according to the protocol described in detail by Needham *et al.*[1] Stock solutions for lasing was prepared by dissolving the solid into dimethyl sulfoxide (DMSO) at 1M concentration. Then it was diluted to 78.4 mM with distilled water.

**Double-stranded calf thymus DNA (type I)** (named ctDNA duplex in the text) was purchased from Sigma Aldrich. ctDNA was dissolved in distilled water to the final concentration of 40.3 mM (12.2 mg/ml). The concentrations of ctDNA solutions were assessed per average mass of a single base (330 g/mol). DNA was mixed with dyes in volume ratios 3:2 (DNA:dye) for cavity lasing experiments and 20:1 for ASE measurements.

**Fragmentation of DNA** by ultrasonication was done using Hielscher UP50H with frequency of 30 kHz. Sonication conditions were done in 5 seconds cycles whereby the sample was first exposed to ultrasounds for 5 s and then 5 s break was applied. The amplitude was adjusted to 100%[2].

**Aggregation of DNA** was done by melting DNA duplex to 92°C. Next the sample was cooled down to 75°C. Divalent magnesium salt was adjusted in heated DNA 100 mM $MgCl_2$. The sample was took off from the heating block and vortexed for 10 seconds[3].

**UV-Vis Spectroscopy**: Absorption spectra were recorded on a CARY-5000 spectrophotometer.

**Steady-state fluorescence spectroscopy**: For recording fluorescence spectra samples were excited with the light of a xenon arc lamp passing through a monochromator with the central wavelength and

transmission bandwidth set to 400 nm and 1 nm, respectively, and a short-pass absorption filter (absorption edge at 450 nm). The emitted light was analysed with a SpectraPro 150 Czerny-Turner imaging monochromator equipped with a CCD camera (Andor DU420A-BU2). The fluorescence light was collected at the right angle to the direction of the excitation light. The cuvette with the solution was oriented at $90^o$ and the films were oriented at approx. $45^o$ angle with respect to the excitation and detection directions. A long-pass filter (edge at 430 nm) in front of the monochromator was used to eliminate the scattered excitation light. The recorded spectra were corrected for the transmission curve of this filter.

**Fabry-Perot cavity lasing.** Lasing spectra in liquid samples were recorded using femtosecond laser working at 0.5 kHz repetition rate and providing energy of a pulse 400 µJ. The output beam was at 800 nm and was tunned in the optical amplifier to spectral range 390-490 nm to adjust the appropriate wavelength for cavity mirrors parameters and sample excitation spectrum. The experimental setup used for lasing experiments is shown in Fig. S1. The mirrors had nearly 100% transmission at 400-405 nm. The reflectance in range 470-570 nm was around 95-99%, centered at 520 - 530 nm.

Examination of lasing in wavelength range 390 – 490 nm indicated that excitation at 430 nm was optimal because of high transmission rate of cavity mirrors at that wavelength and well matching with the absorption maximum of dye molecules intercalated between the DNA strands[4]. The cavity thickness was in range 2 µm – 6 µm for dye in solvents. In case of dye stained DNA the cavity thickness was 6 – 10 µm.

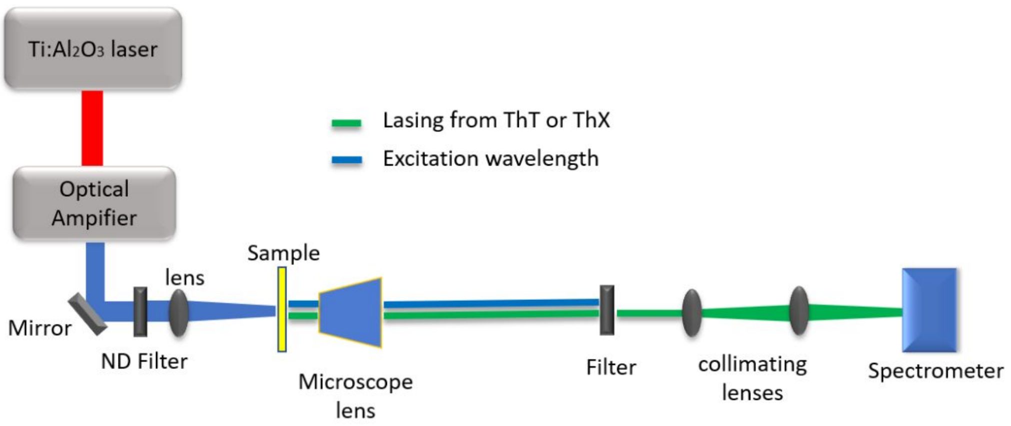


**Scheme S1.** Schematic view of the experimental setup used for studies of the Fabry-Perot cavity lasing.

The lasing signal from the gain medium in cavities was collected parallel to the direction of the excitation beam. Blue light filter was removing light >470 nm and only the lasing signal from the sample was reaching the detector. Lasing was collected with a Pixes spectrometer, whose spectral resolution was around x nm. For the determination of lasing thresholds the intensity of the excitation light was gradually increased by stepwisely moving the grey filter using the Thorlabs motor system and the spectrum of the emitted light was simultaneously monitored in the detector. Plots of the dependence of the pump energy and emission were used to determine the lasing thresholds.

**Amplified spontaneous emission (ASE) in solid films** was measured using 5 kHz Ti:Sapphire femtosecond amplifier system (Legend Elite Duo) with a beta-barium borate (BBO) crystal that was used to produce 400 nm of pulse energy 300 µJ. The beam was first expanded with a telescope formed by a pair of spherical

lenses with focal lengths of -50 and 125 mm. Then it was focused with a cylindrical lens in order to form a narrow stripe of light at the position of the film sample.

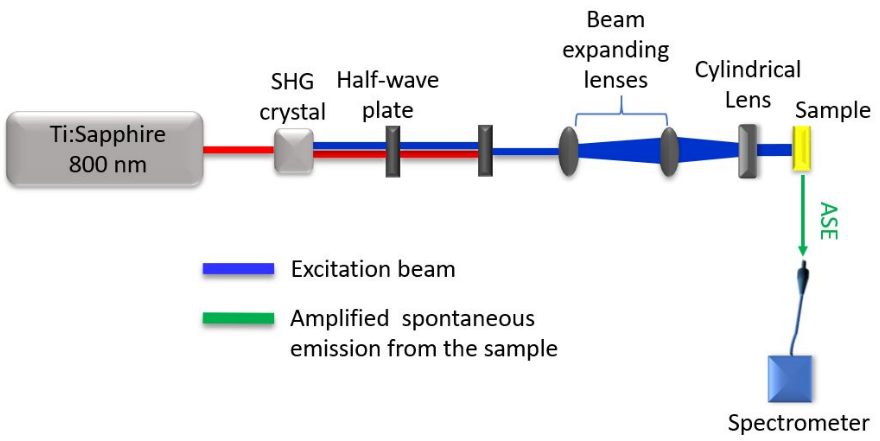


**Scheme S2.** Schematic view of the experimental setup used for studies of ASE in films.

The studied films were oriented perpendicularly to the direction of the excitation beam. The amplified spontaneous emission was detected in the plane of the films. The ASE light was collected with a fiber and delivered to the Ocean Optics USB 2000 spectrometer, whose spectral resolution was around 4 nm. For the determination of ASE thresholds the intensity of the excitation light was gradually increased by rotating the half-wave plate and the spectrum of the emitted light was simultaneously monitored.

## Additional graphs

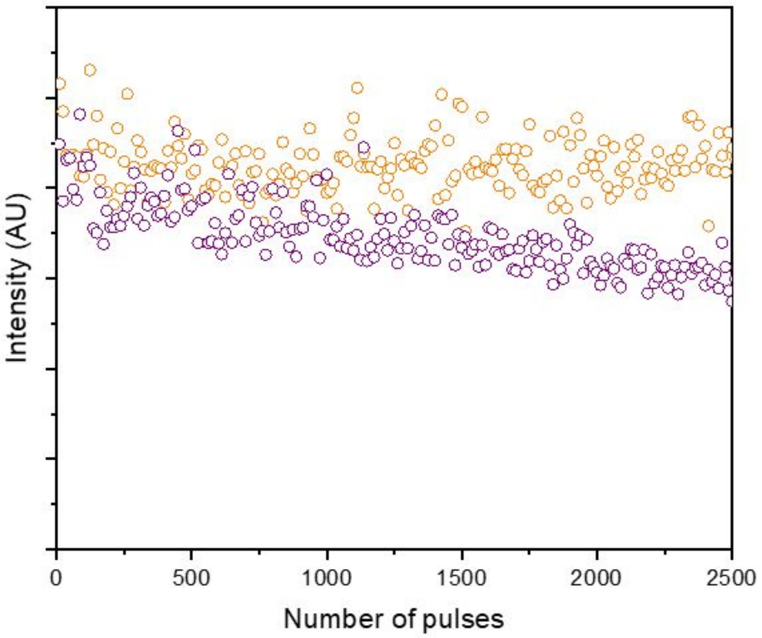


**Fig. S1.** Photodegradation experiment of ThT dissolved in ethanol (violet open circles) and butanol (orange open circles. Samples were excited at 430 nm.

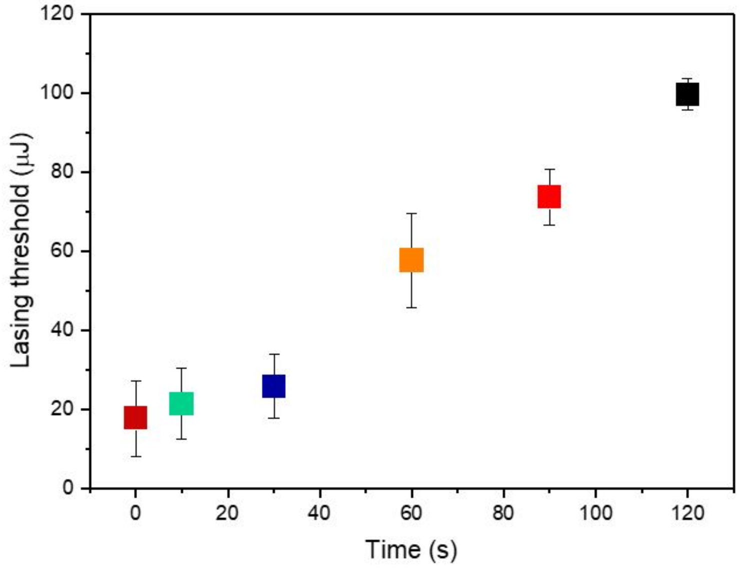


**Fig. S2.** Lasing thresholds measured in ThT-stained DNA exposed to ultrasonication for a specific time. Ultrasonication was performed in intervals: 5 seconds sonication on and 5 seconds sonication off with amplitude adjusted to 100. The squares of different colors with error bars represent the three consecutive measurements used to determine the average lasing threshold. Samples were excited at 430 nm.

References


1. Needham, L.-M.; Weber, J.; Varela, J. A.; Fyfe, J. W.; Do, D. T.; Xu, C. K.; Tutton, L.; Cliffe, R.; Keenlyside, B.; Klenerman, D., ThX–a next-generation probe for the early detection of amyloid aggregates. *Chemical Science* **2020,** *11* (18), 4578-4583.

2. Sambrook, J.; Russell, D. W., Fragmentation of DNA by sonication. *Cold spring harbor protocols* **2006,** *2006* (4), pdb. prot4538.

3. Bui, V. C.; Nguyen, T. H., DNA aggregation induced by Mg2+ ions under different conditions. *Journal of Molecular Recognition* **2018,** *31* (9), e2721.

4. Hanczyc, P.; Rajchel-Mieldzioc, P.; Feng, B.; Fita, P., Identification of thioflavin T binding modes to DNA: a structure-specific molecular probe for lasing applications. *The Journal of Physical Chemistry Letters* **2021,** *12* (22), 5436-5442.
